# Novel direct effect of CCR2 receptor on follicle activation process

**DOI:** 10.3389/fendo.2025.1613270

**Published:** 2025-08-01

**Authors:** Yamila Gamaleri, Delfina Sol Ferman, Alison Ting, Eduardo Raúl Dascal, Alejandro Lomniczi, Juan Pablo Jaworski, Marina Cinthia Peluffo

**Affiliations:** ^1^ Centro de Investigaciones Endocrinológicas “Dr. César Bergadá” (CEDIE) -CONICET–FEI–División de Endocrinología, Hospital de Niños “Ricardo Gutiérrez”, Buenos Aires, Argentina; ^2^ Expanse Bio, LLC, Charleston, SC, United States; ^3^ Department of Obstetrics, Gynecology & Reproductive Sciences, University of California, San Diego, San Diego, CA, United States; ^4^ Department of Physiology and Biophysics, Faculty of Medicine, Dalhousie University, Halifax, NS, Canada; ^5^ National Institute of Agricultural Technology-Instituto Nacional de tecnología agropecuaria (INTA)-CONICET, Castelar, Argentina

**Keywords:** follicle activation, CCR2, MCP-1, CCL2, folliculogenesis, feline

## Abstract

**Introduction:**

The regulation of primordial follicle activation is crucial for maintaining ovarian function, the duration of the reproductive phase, and fertility in women; therefore, we propose as our general objective to determine the physiological role of the chemokine receptor CCR2 within the follicular activation process.

**Methods:**

Ovarian cortex fragments from adult domestic cats (*Felis catus*) were cultured under different experimental groups: control (media alone), CCR2 antagonist (1µM), and recombinant chemokine CC-motif ligand 2 (CCL2) at two concentrations (10 ng/ml and 100 ng/ml) for 4 h or 48 h. At the end of the culture, the fragments were collected for RNA extraction, cDNA synthesis, and quantitative real-time PCR (4 h) or fixed and processed for paraffin embedding (48 h) for hematoxylin and eosin staining or immunohistochemistry for Ki67, bromodeoxyuridine (BrdU) and AKTp.

**Results:**

Stimulation of CCR2 significantly increased the normalized mRNA expression of *KIT, FOXO3* (10 ng/ml), and *AKT* (100 ng/ml) compared to the control (p<0,05). Moreover, there was a significant increase in the percentage of transitional follicles (and a decrease in primordial follicles), together with an increase in oocyte diameter compared with the control and the antagonist groups (p<0.05). Also, in the presence of CCL2, a higher proportion of transitional and primary follicles immunolabeled for BrdU and Ki67 (p<0.05), as well as intense AKTp staining in the nucleus and cytoplasm of oocyte and granulosa cells of primordial, transitional and primary follicles, were observed. On the contrary, a lower proportion of BrdU and Ki67-positive follicles were observed in the antagonist group (p < 0,05).

**Conclusion:**

Our results show a direct effect of the chemokine CCL2 and a role of the CCR2/CCL2 system on the ovarian cortex, suggesting that the CCR2 receptor signaling in the ovarian cortex may regulate events critical for promoting the stimulation of the transition from primordial to primary follicles.

## Introduction

In most mammals, before or after birth, the ovogonia are transformed into primary oocytes to form primordial follicles. Primordial follicles constitute the resting follicle pool, and their activation is an irreversible process characterized by morphological modification of pre-granulosa cells (from flat to cuboidal), resumption of cell proliferation, and initiation of oocyte growth (1–4). Interestingly, the molecular factors and mechanisms responsible for the control of the onset of folliculogenesis are poorly understood in many species and remain one of the significant unknowns in ovarian biology.

A rodent study has shown that primordial follicle activation depends on the mTORC1-KITL system in pre-granulosa cells and its receptor KIT-PI3K signaling pathway in the oocyte (5). PI3K activation induces protein kinase B (Akt) phosphorylation and translocation of the transcription factor FOXO3 from the nucleus to the cytoplasm, activating primordial follicles (6–8). FOXO3 knock-out mice exhibit global activation of primordial follicles, resulting in premature depletion and infertility (9, 10). In contrast, a recent report suggested that the FOXO3 signaling pathway may not be responsible for the activation process in primates (11). The mentioned discrepancies highlight the importance of studying these processes in different species. Indeed, in mammals (except rodents), FOXO3 expression has not been observed in primordial follicle oocytes (12). The regulatory molecule(s) upstream of the mTORC1 signaling pathway remains unknown, even in rodents.

Preliminary results in our laboratory, as well as others, indicate that chemokines may be involved in the regulation of the primordial follicle activation process. We have demonstrated IHC positive labeling of the chemokine receptor CCR2 and its ligands C-C Motif Ligand 2 (CCL2, also known as/MCP1) and Monocyte Chemotactic Protein-2 (MCP2) in paraffin-embedded cat ovarian sections (13, 14), observing a differential and highly variable marking in primordial follicles. Interestingly, while the expression of CCR2 and its ligand chemokines was intense in the oocyte’s nucleus and/or cytoplasm in many feline primordial follicles, it was absent in others. Similar results were obtained from rhesus monkey ovarian sections. We speculate that this differential expression may be associated with the activation process of primordial to primary follicles. Supporting this idea, previous studies in murine neonatal ovaries have shown that the interaction of another chemokine receptor (CXCR4) and its ligand (SDF1) inhibited the transition from primordial to primary follicles (15). Also, a recent study speculates that in rodents, the chemokine receptor CCR2 plays a role in follicular activation, although their results do not demonstrate such an assumption (16).

Regulation of the activation of quiescent ovarian follicles is crucial for maintaining ovarian function, duration of the reproductive stage, and fertility in women. Activation of primordial follicles is a natural process of primary follicle depletion, leading to reproductive senescence. This process is accelerated in some cases, such as primary ovarian insufficiency or premature ovarian failure. Therefore, a better understanding of the molecular mechanisms involved in follicular activation could contribute to elucidating the causes of infertility and develop better treatment strategies.

Therefore, this study aims to determine the physiological role of the CCR2 chemokine receptor in the follicular activation process. Evaluating whether stimulation or inhibition of the CCR2 chemokine receptor impacts the expression of genes associated with primordial follicle activation. In turn, we will determine whether stimulation (CCL2) or inhibition (CCR2-specific antagonist) of the CCR2 chemokine receptor promotes or inhibits follicular activation (i.e., altered gene expression, morphological modifications of the pre-granulosa cells (from flat to cuboidal) and reinitiation of cell proliferation, etc.).

## Materials and methods

### Animals and ovary collection

Ovaries from adult domestic cats (Felis catus; n=49) at different stages of the natural estrous cycle during the breeding season were used. They were donated by the “Centro de Salud Animal de la Municipalidad de Merlo” (Province of Buenos Aires, Argentina) after routine spaying procedures. After surgery, extracted ovaries were transported to our laboratory in refrigerated saline solution within 2–3 h and processed immediately. Ovaries presented stigmas, as an indication of recent ovulation, or with cysts were discarded. Ethical approval was not required as discarded material from a shelter was used.

### Ovarian cortex tissue culture

Small fragments of feline ovarian cortex were isolated as described by Ting et al. (11). Briefly, the ovaries were cleaned and defatted by trimming. 0.5-mm-thick outermost ovarian cortical pieces were collected using a tissue slicer (Thomas^®^Stadie-Riggs) and 1x1 mm sections were made using a scalpel. Only tissues with visible follicles (at least one primordial and one primary follicle) under a high-resolution trinocular stereo microscope equipped with a digital camera (Nikon, SMZ-18) and a thermal stage (Nikon-Tokai, Mats-4020W) were included for culture. Ovarian fragments were randomly placed on cell culture inserts (3–4 pieces/well) 0.4 µm pore size (Millipore) in a culture medium (αMEM supplemented with 0.3% (v/v) BSA, 0.5 mg/ml bovine fetuin, 5 μg/ml transferrin, 0.5 μg/ml insulin, and 5 ng/ml selenium) in the presence or absence of the different treatments (3 wells/group). **Supplementary Table 1** depicts the fragment area and follicle density among the different treatments. The fragments were incubated for 4 h (gene expression analysis) or 48 h [histological analysis and steroid hormone determination with and without BrdU (50 mM)] at 38°C and 5% CO2 in the following experimental groups: (i) media alone (control group); (ii) media plus a CCR2-selective antagonist (1 µM); (iii) media plus CCL2–10 ng/ml (Life Technologies); (iv) media plus CCL2–100 ng/ml. CCL2 concentrations were chosen based on literature and preliminary results. We used “RS-504393” as a CCR2-antagonist (Tocris Biosciences) since it has proven to selectively antagonize the CCR2 receptor (IC50: 98 nM and > 100 μM for inhibition of human CCR2 and CCR1 receptors, respectively) (17), and has already been used by us in cumulus-oocyte complexes (COCs) cultures (18, 19). At the end of the culture, fragments were either stored at -80°C for subsequent RNA extraction (4 h cultures) or fixed in 4% paraformaldehyde (PFA) at 4°C (ON, “overnight”) for further processing and histological analysis (48 h cultures with and without BrdU). In addition, culture medium (48 h) was stored at -80°C for subsequent measurement of steroid hormones [Progesterone (P4) and Estradiol (E2)] to ensure the absence of large follicles (such as antral follicles). The steroid content was analyzed (Shown as **Supplemental Figure 1**) by the Endocrinology Laboratory of the CEDIE “Hospital de Niños Ricardo Gutiérrez” using a COBAS e411 analyzer, an automated clinical platform based on electrochemiluminescence (Roche Diagnostics GmbH) (20–22).

### mRNA extraction and gene expression analysis

After RNA extraction following the Trizol extraction method (23), single-stranded cDNA was synthesized from total RNA (1µg) using the High-Capacity cDNA Reverse Transcription Kit (Applied Biosystems) as described previously (18, 19, 22). Afterward, qPCR was performed for key genes associated with the follicle activation process (*PIK3Ca, FOXO3, KIT, KITL, AKT, and mTOR*). For this purpose, specific primers (Forward and Reverse) were designed for each gene, according to the *Felis catus* sequences published in the National Center for Biotechnology Information (NCBI). Relative gene expression levels were determined using a standard curve of target genes and normalized to ribosomal RNA protein 18S and Glyceraldehyde-3-phosphate dehydrogenase (*GAPDH*) levels. For that purpose, a pool of samples (representatives of all treatment groups) was 5-fold titrated and ran in each tested plate. Each primer pair concentration was optimized independently: *GAPDH, 18s, KITL, AKT, and mTOR* primers were used at 250 nM; *FOXO3* at 100 nM; and P*IK3C*a and *KIT* at 500 nM. Each reaction consisted of 5 µl of Master Mix (FastStart Universal SYBR Green Master, Roche), 2 µl of primer mix, and 3 µl of cDNA or H20 (blank, NTS) per well, with the following cycling conditions: 10 min at 95°C, and 45 cycles of 15 sec at 95°C and 1 min at 60°C. Melting curves were performed to corroborate qPCR signal specificity by a single peak at the desired melting temperature (Tm). In addition, internal positive and negative controls were used to assess the validity of each plate. The list of primers, with the accession numbers of each target sequence, is listed in **Supplementary Table 2**.

### Tissue processing and immunohistochemistry

Fragments were fixed in a 4% PFA at 4°C ON. After fixation, tissue fragments were processed for paraffin sectioning (3-5 µm), serial sections were stained for hematoxylin and eosin (H&E) and used for IHC.

IHC was performed as previously published (11, 24). Cortex fragments were incubated with the different primary antibodies (Ki67 1:50, AKTp 1:50, and BrdU 1:200) in a humid chamber at 4°C ON. The primary antibodies were detected by adding a second biotinylated universal antibody (rabbit/mouse anti-IgG made in horse, dilution 1:400) and the Vectastain ELITE ABC (Universal) Kit (Vector Cat # PK-6200), stained with diaminobenzidine (Dab peroxidase substrate (Vector Cat # SK-4100) and counterstained with hematoxylin. Negative controls (lacking primary antibodies) were processed on adjacent tissue sections.

### Quantification of primordial and primary follicles in tissue sections

After staining according to the method of Edna Prophet (1992) (25), morphology, number, and size of follicles at different stages of development (primordial, primary, and secondary) and the different cell types observed in each ovarian cortex fragment were analyzed. All fragments were individually analyzed under the microscope to determine the proportion of primordial, primary, and secondary follicles as an indicator of follicular development. Developmental classification of follicles was made based on their morphological characteristics: i) primordial follicles (20-30 µm in diameter) with a single layer of flattened granulosa cells; ii) transitional follicles (30-50 µm) containing at least one cuboidal granulosa cell and a growing oocyte, iii) primary follicles (30-50 µm) containing a whole layer of cuboidal granulosa cell and a growing oocyte, and iiii) secondary follicles (100-200 µm) comprising more than one layer of cuboidal granulosa cells (26, 27).

### Statistical analysis

Experiments were performed in triplicate or quadruplicate. The normal distribution of the results was evaluated, and if the data did not follow a normal distribution, they were transformed using log+1 or log+10. When several groups were assessed, analysis of variance (ANOVA) was used, followed by the Newman-Keuls test using GraphPad Prism 5 software (GraphPad Software, Inc., San Diego, CA, United States). On the other hand, when evaluating differences in rates/proportions, Chi-Square and/or Fisher exact tests were used. In all cases, a significant difference was considered when p-value <0.05.

## Results

### CCL2 treatment increases key gene expression of the follicular activation process

**Figure 1:**
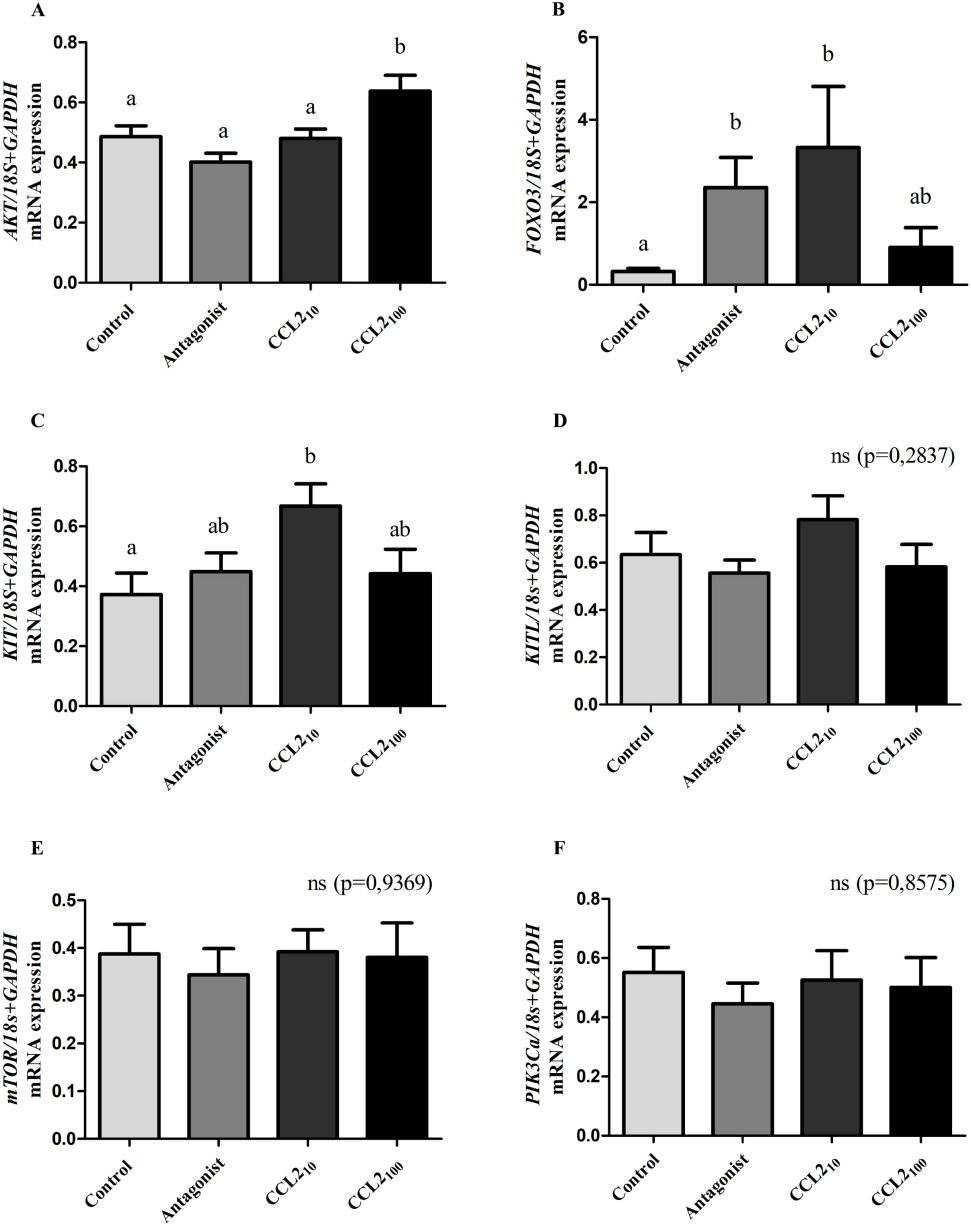
Expression of key genes in the follicular activation process in feline ovarian cortex fragments. CCL2 increased the expression of key genes (*AKT*, *FOXO3*, and *KIT*) in the follicular activation process. Normalized mRNA expression levels (mean ± SEM; n=9 fragments/treatment, from 3 different cultures) of *AKT* **(A)**, *FOXO3* **(B)**, *KIT* **(C)**, *KITL* **(D)**, *mTOR* **(E)** and *PIK3Ca* **(F)** in feline ovarian cortex fragments cultured 4 h in the presence of different treatments (Control, CCR2 Antagonist, CCL2_10_ and CCL2_100_) assessed by qRT-PCR. 18s rRNA and *GAPDH* served as invariant controls for normalization. Significant differences between groups are represented by different letters (ANOVA; p<0.05).

Normalized RT-qPCR showed a significant increase in *AKT, FOXO3*, and *KIT* mRNA levels in the CCL2 group compared to the control group (**Figures 1A-C**, p<0.05; ANOVA). The increase in *AKT* expression occurred at the highest dose (100 ng/ml) of CCL2 (from now CCL2_100_), whereas, for *FOXO3 and KIT*, it was observed at the lowest dose (10 ng/ml, from now CCL2_10_). Significant differences were also observed for *AKT* with CCL2_100_ relative to all groups.

Additionally, we observed a significant increase in *FOXO3* mRNA expression in the presence of the CCR2-antagonist compared to the control group (**Figure 1B**, p<0.05). On the other hand, normalized expression levels of *KITL, mTOR, and PIK3Ca* genes did not yield significant differences amongst all groups (**Figures 1D–F**, p>0.05).

### CCL2 treatment increases the number of transitional and primary follicles and the oocyte diameter

**Figure 2:**
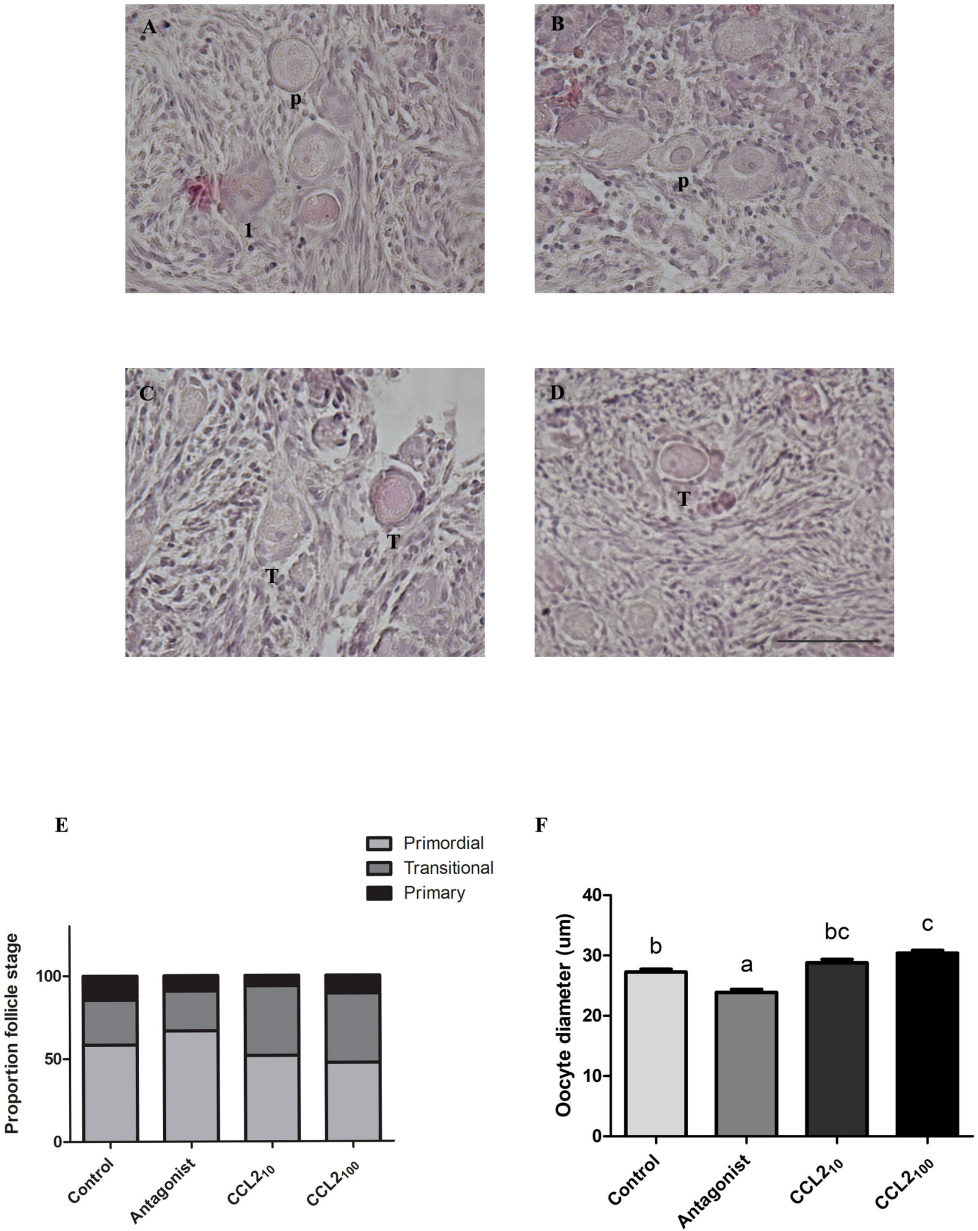
Percentage of primordial, transitional and primary follicles together with oocyte diameter in feline ovarian cortex fragments. CCL2 increased the proportion of transitional follicles and the oocyte diameter in feline ovarian cortex fragments *in vitro*. Histological sections of feline ovarian cortex fragments that were cultured 48 h in the presence of different treatments [Control **(A)**, CCR2 Antagonist **(B)**, CCL2_10_ **(C)**, and CCL2_100_ **(D)**], stained with H&E. The presence of primordial follicles (P), transitional (T) and primary follicles (1) is indicated in the different photographs. The photographs were taken using the 20x objective of the microscope. Scale bars 50 μm. Panel **(E)** shows the proportion of primordial (light gray), transitional (gray) and primary (black) follicles in each of the experimental groups (n=12–14 fragments/group, from 4 cultures). Significant differences were observed between the proportions in the different groups (Chi-square 4 groups, 0.0028). Panel **(F)** shows oocyte diameter (µm) in each experimental group (n=113–140 follicles/group, from 4 cultures). Significant differences between groups are represented by different letters (ANOVA; p<0.05).

Following 48 h culture under four different conditions (media alone, media/antagonist, media/CCL210 and media/CCL2100), feline ovarian cortex fragments were fixed and processed to generate histological sections stained with H&E to evaluate the effect of the different treatments on follicle activation (i.e., transition to primary follicular stage, increase in oocyte diameter, etc.). At least one primordial, one transitional and one primary follicle were identified in each tissue, while secondary follicles were sporadically observed in few slices and excluded for further analysis. **Figure 2** shows representative images of H&E-stained cortex fragments from different experimental groups, where primordial (p), transitional (t) and primary (1) follicles can be observed. The proportion of primordial, transitional and primary follicles within each fragment from the total number of follicles in each group was assessed, and the results are plotted in panel E of **Figure 2**. A significant difference in the proportion of primordial, transitional and primary follicles amongst the different treatments was found (**Figure 2E**, p=0.0028, Chi-square). Also, when analyzing the proportion of primordial or transitional (against the rest of the follicles) amongst the different treatments (Fisher: p= 0.0110 and 0.0013, respectively). In the CCL2_10_ group, the proportion of transitional follicles (42%) was significantly higher compared to the control group and the CCR2-antagonist (p=0.0122 and p=0.0029, respectively). At the higher concentration, the CCL2_100_ group also shows an increased proportion of transitional follicles compared to both control and the antagonist groups (p=0.0135 and p=0.0034, respectively).

When the oocyte size (as another activation indicator) was measured, CCL2 and the CCR2-antagonist had opposite effects on the oocyte diameter (**Figure 2F**). CCL2_100_ led to an increased oocyte diameter compared to the control, whereas the antagonist decreased it (p<0,001; ANOVA). CCL2_10_ also showed a trend of increased oocyte diameter, although the difference was not statistically significant.

### CCR2-antagonist reduces cell proliferation in transitional and primary follicles

**Figure 3:**
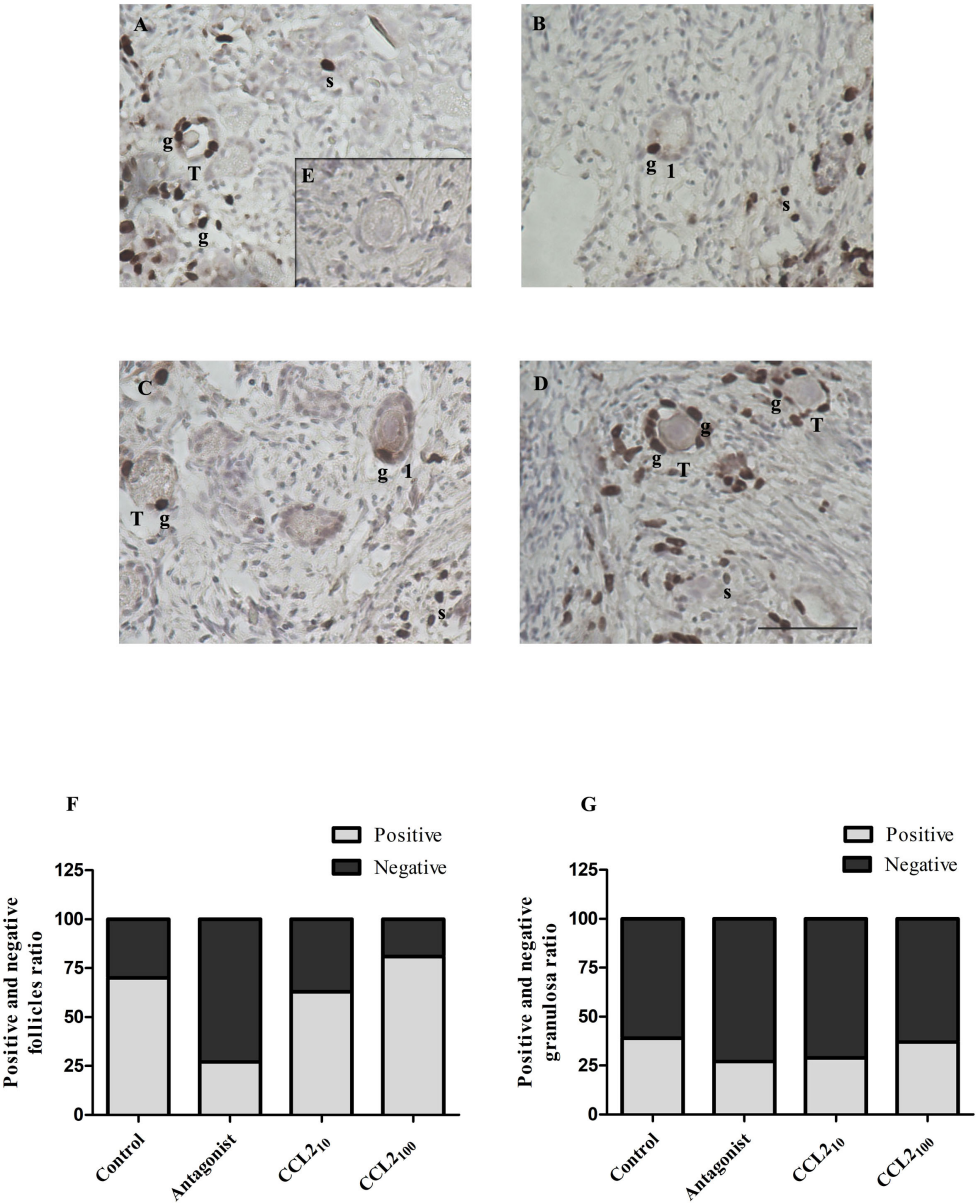
BrdU staining in feline ovarian cortex fragments. A higher proportion of BrdU-positive follicles in the CCL2-treated group and a lower proportion of positive ones in the presence of the selective CCR2 antagonist. Histological sections of feline ovarian cortex fragments that were cultured for 48 h in the presence of different treatments [Control **(A)**, CCR2 Antagonist **(B)**, CCL2_10_ **(C)**, and CCL2_100_ **(D)**], subjected to IHC for BrdU. Positive immunostaining (brown) with varying intensities can be seen in the different panels in the nuclei of granulosa cells (g) from transitional and primary follicles and in stromal cells (s) (n= 6–7 fragments/group, from 3 cultures). The negative control (incubated without the first antibody, panel **E**) was not positively labeled. Photographs were taken using a 20x objective. Scale bars 50 μm. Panels **(F, G)** show the proportion of transitional and primary follicles or granulosa cells with positive and negative immunostaining, respectively. Significant differences were observed between the proportions of positive and negative follicles among the different groups (Chi-square 4 groups, p= 0.0267).

**Figure 4:**
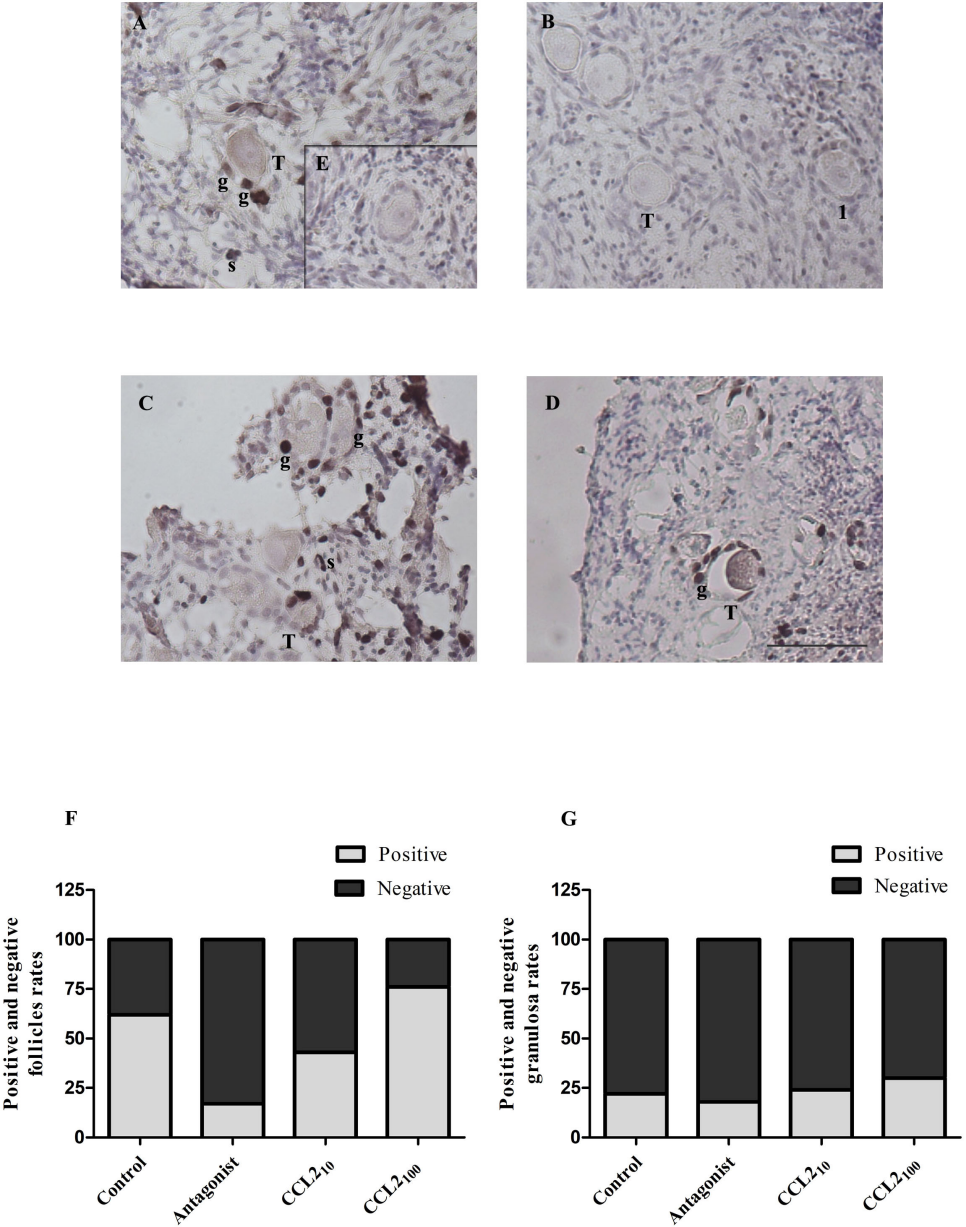
Ki67 staining in feline ovarian cortex fragments. A higher proportion of Ki67 positive follicles in the CCL2-treated group and a lower proportion of positive ones in the presence of the selective CCR2 antagonist. Histological sections of feline ovarian cortex fragments that were cultured for 48 h in the presence of different treatments [Control **(A)**, CCR2 Antagonist **(B)**, CCL2_10_ **(C)**, and CCL2_100_ **(D)**], subjected to IHC for Ki67. Positive immunostaining (brown) with varying intensities can be observed in the different panels in the nuclei of granulosa cells (g) from transitional and primary follicles and stromal cells (s) (n= 4–5 fragments/group, from 2 cultures). The negative control (incubated without the first antibody, panel **E**) was not positively labeled. Photographs were taken using the 20x objective. Scale bars 50 μm. Panels **(F, G)** show the proportion of transitional and primary follicles or granulosa cells with positive and negative labeling, respectively. Significant differences were observed between the proportions of positive and negative follicles among the different groups (Chi-square 4 groups, p= 0.0024).

Cellular proliferation in follicles was assessed by the incorporation of BrdU (**Figure 3**) and IHC detection of Ki67 (**Figure 4**). Positive staining for both was observed in the nuclei of stromal and granulosa cells of transitional, primary and secondary follicles.

The proportion of transitional and primary follicles (**Figure 3F**) or granulosa cells (**Figure 3G**) that were positively or negatively labeled for BrdU was analyzed among the groups. The majority (92%) of the positive follicles correspond to the transitional category. A significant difference in the proportion of positively labeled transitional and primary follicles was observed within the ovarian cortex when all groups were analyzed (p= 0.0267, Chi-square). CCR2-antagonist significantly reduced the proportion of BrdU-positive transitional and primary follicles (25%) compared to the control (70%) (p=0.0251), CCL2_10_ (60%) (p=0.0359) and CCL2_100_ (80%) (p=0.0014). Interestingly, the highest proportion of BrdU-positive transitional and primary follicles was detected in the CCL2_100_ group. Although the proportion of BrdU-positive transitional and primary follicles in the CCL2_100_ group was higher than in the control group (80% vs. 70%, respectively), they were not significantly different. In contrast, no difference was observed in the proportion of BrdU-positive cells when the results were analyzed based on granulosa cell staining amongst all groups (**Figure 3**, p>0.05).

The proportion of Ki-67 positive and negative transitional and primary follicles (**Figure 4F**) or granulosa cells (**Figure 4G**) amongst the four groups showed a similar pattern to those described for BrdU, denoting a significant difference in the proportion of positively labeled transitional and primary follicles (p= 0.0024, Chi-square). The majority (92%) of the positive follicles correspond to the transitional category. The proportion of Ki67-positive transitional and primary follicles in the control, CCL2_10,_ and CCL2_100_ groups were 60%, 45%, and 75%, respectively. The lowest proportion of Ki67-positive follicles was observed in the CCR2-antagonist group (20%). This reduction was statistically significant compared to the control (**Figure 4F**, p=0.0050) and the CCL2_100_ groups (**Figure 4F**, p=0.0006). On the other hand, the proportion of Ki67-positive granulosa cells was similar in all the groups (**Figure 4G**, p>0.05). No positive staining was observed without the primary antibody (internal negative control for Brdu and Ki67 IHC; panel E of **Figures 3** and **4**, respectively).

### Intense AKTp labeling in the nucleus and cytoplasm of oocyte and granulosa cells of primordial, transitional and primary follicles in the presence of CCL2.

**Figure 5:**
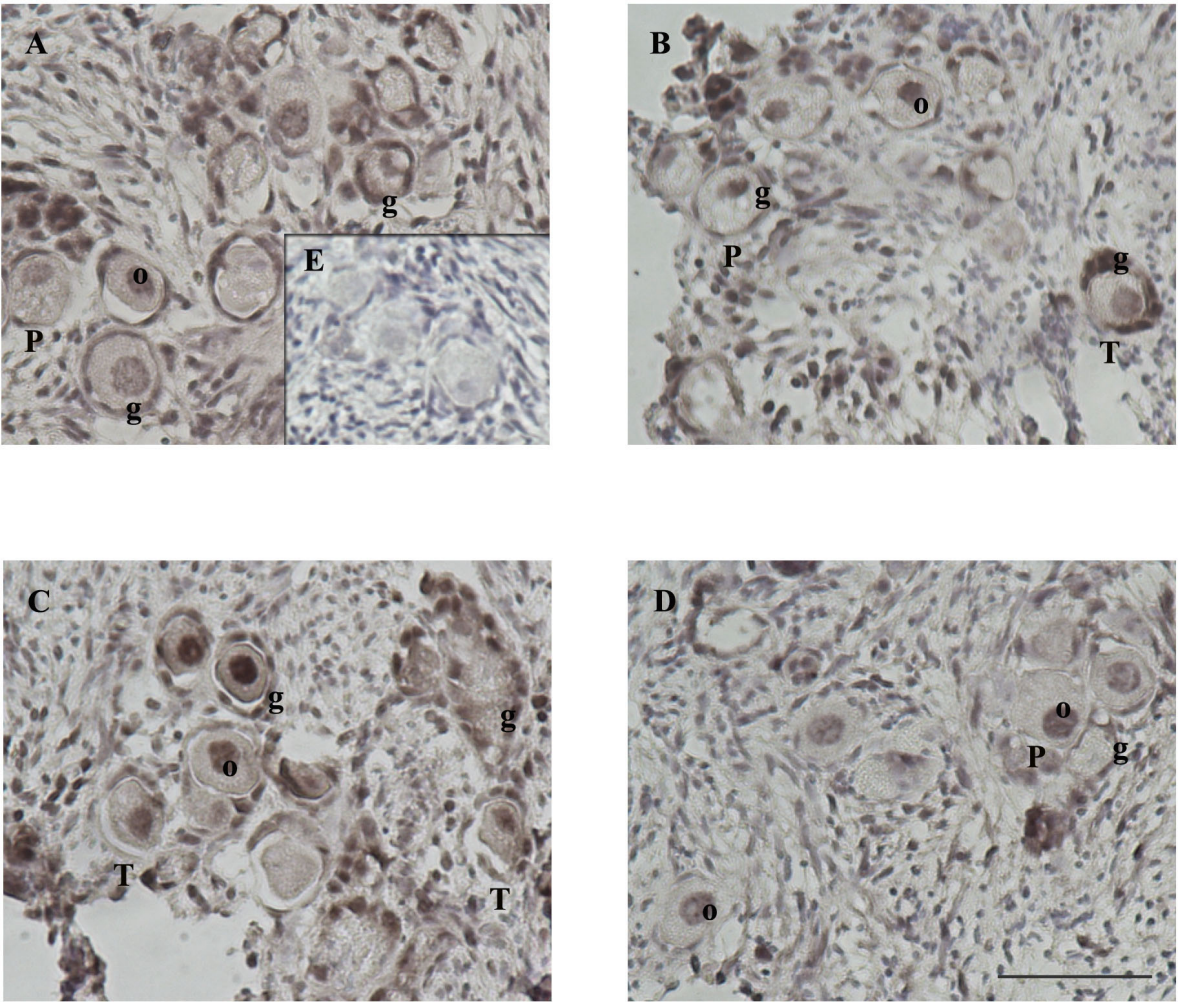
AKTp staining in ovarian cortex fragments. Intense AKTp labeling in the nucleus and cytoplasm of oocyte and granulosa cells of primordial, transitional and primary follicles in the presence of CCL2. Feline ovarian cortex fragments were cultured for 48 h in the presence of different treatments [Control **(A)**, CCR2 Antagonist **(B)**, CCL2_10_ **(C)**, and CCL2_100_ **(D)**], subjected to IHC for AKTp. Positive immunostaining (brown) with varying intensities can be observed in the cytoplasm or nucleus of the oocyte (o) and granulosa cells (g) of primordial (P), transitional (T) and primary (1) follicles in the different panels. Positive labeling was also observed in stromal cells (n= 4–5 fragments/group, from 2 cultures). The negative control (incubated without the first antibody, panel **E**) did not show immunoreactivity. Photographs were taken using a 20x objective. Scale bars 50 μm.

AKTp-positive staining was observed in the stroma, granulosa cells, and oocyte of primordial, transitional, primary, and secondary follicles. AKTp localization was observed in the cytoplasm and/or the nucleus. **Figure 5** shows representative images of positive staining (brown color, panels A-D) within histological sections of feline ovarian cortex fragments cultured for 48 h under different experimental conditions. Remarkably, CCL2_10_-treatment resulted in very intense AKTp staining in granulosa cells and the nucleus and cytoplasm of the oocyte, both in primordial, transitional and primary follicles (**Figure 5C**). Positive immunostaining was observed in all the treatments. No staining was detected in the negative internal control without primary antibody (**Figure 5E**). **Table 1** shows the degree of staining subjectively graded as well as cellular localization of the positive immunolabeling that was further categorized within the different compartments (granulosa cells and oocyte only, or both) of primordial, transitional and primary follicles present in the cortex fragments, observing a different pattern of positive immunolocalization.

**Table 1:** AKTp immunohistochemistry results within feline ovarian cortex fragments that were cultured 48 h in the presence of different treatments (Control, CCR2 Antagonist, CCL2_10_, and CCL2_100_).

Cellular localization	Control				CCR2 Antagonist					CCL2_10_			CCL2_100_
Primordial, transitional, and primary follicles	+				+					+++			+
Ovarian stroma	+/-				+					++			+/-

Follicles	Primordial				Transitional					Primary			
Cellular localization	Control	CCR2 Ant	CCL2_10_	CCL2_100_	Control	CCR2 Ant	CCL2_10_	CCL2_100_		Control	CCR2 Ant	CCL2_10_	CCL2_100_
Oocyte	x	x	x	x	-	-	-	-		-	-	-	-
Granulosa	x	x	-	x	x	x	xx	xxx		xxx	-	xx	x
Both	xxx	xxx	xxx	xx	xxx	xxx	xxx	xx		x	-	xx	xxx

The degree of staining was graded subjectively as very strong (+++), strong (++), positive (+), or weak/varied (+/−). Proportion of cellular localization of the positive immunolabeling was further categorized within the different compartments (granulosa cells and oocyte only, or both) of primordial, transitional, and primary follicles present in the cortex fragments, designating as high (xxx), intermediate (xx), low (x), or null (-).

## Discussion

This study focused on studying whether stimulation or inhibition of the CCR2 chemokine receptor in ovarian cortex fragments was able to impact processes related to follicle activation (I.e., expression of key genes, morphological modifications of pre-granulosa cells (from flat to cuboidal), the restart of cell proliferation and the initiation of oocyte growth).

We observed significant differences in the expression of three of the six key genes in this process. In the presence of recombinant chemokine CCL2, a substantial increase in normalized mRNA expression *of KIT, FOXO3* (CCL2_10_), and *AKT* (CCL2_100_) was observed compared to the control group, supporting the idea that CCR2 receptor activation would stimulate the follicular activation process. A previous study showed that primordial follicle activation depends on the mTORC1-KITL system in pre-granulosa cells and its receptor KIT-PI3K signaling pathway in the oocyte (5) in rodents. The binding of KITL to its receptor results in the phosphorylation of KIT, which activates the PI3K/AKT/FOXO3 signaling pathway (28). Likewise, according to the results, the CCL2 ligand activates CCR2 in various cell types, triggering several signaling pathways (29–32), including the PI3K/AKT/mTOR pathway. This has been shown to lead to primordial follicle activation (33–35). PI3K activation induces Akt phosphorylation and translocation of the transcription factor FOXO3 from the nucleus to the cytoplasm (becomes inactivated), resulting in primordial follicle activation (6, 7, 28). On the other hand, *FOXO3* knockout mice exhibit global activation of primordial follicles, resulting in premature follicular depletion and subsequent infertility (9, 10), while overexpression of constitutively active FOXO3 increases ovarian reproductive capacity (36). Moreover, *FOXO3* knockdown in bovine ovarian cultures has been shown to promote primordial follicle activation (37).

As for *KITL, mTOR, and PI3Kca*, no significant differences were observed in the mRNA expression of these genes between the groups. Although *KITL* mRNA expression showed a similar pattern to that observed for *KIT*, this did not reach significance. This could be due to the heterogeneous nature of the sample. Gene expression was analyzed from the extraction of total RNA from cortex fragments containing varying numbers of primordial, primary, secondary, and perhaps some incipient antral follicles, so it may be that in certain follicles, the expression and activation of the KIT-KITL, mTOR, and PI3Kca system are different. An alternative explanation for this result is that in the mouse, the primordial follicles express KIT and KITL, whereas primary follicles do not express KITL (38). Therefore, the lack of observed effect in the CCL2-treated group can be attributed to greater primary follicles.

Unexpectedly, FOXO3 mRNA expression increased in the presence of CCL2 and with the CCR2 antagonist. This is a seemingly contradictory result, but as mentioned above, as follicles at different stages of development coexist in the cultured fragments at the same time, the CCR2 receptor could play various roles through increasing FOXO3 expression (in follicles at different stages) as FOXO3 is involved in a variety of processes such processes, such as inflammation, metabolism, autophagy, apoptosis, oxidative stress, cell cycle arrest and DNA damage repair among other (39, 40). Thus, it cannot be discarded. For example, it has been demonstrated that FOXO3 is expressed in chicken ovarian tissue, functioning as an initiator of apoptosis in granulosa cells (41). Additionally, FOXO3 has been shown to play a pro-apoptotic role in granulosa cells of early antral follicles (42); therefore, it cannot be ruled out that FOXO3 may also play a similar role in smaller follicles. Numerous studies reported that FOXO3 played a role in follicular development (43–46). Studies in porcine have demonstrated its localization in the oocyte cytoplasm and granulosa cells of primordial, primary, and secondary follicles (47, 48). In rodents, FOXO3 is highly expressed in the oocytes of quiescent primordial follicles (12). However, in most mammals (except for rodents, humans, and pigs), FOXO3 expression in primordial follicle oocytes has not been reported (12, 49). On the other hand, a recent work published by Dr. Ting demonstrated that the signaling pathway involving FOXO3 may not be responsible for the activation process in primates (11), manifesting the differences and importance of studying these processes in different species. It is essential also to note that the biological effects of FOXO3 are primarily determined by its phosphorylation, which has an opposite effect depending on whether it takes place in the nucleus or cytoplasm. Whereas phosphorylation of FOXO3 in the nucleus can promote its translocation into the cytoplasm and inhibit its transcriptional activity, phosphorylation of FOXO3 in the cytoplasm leads to its translocation into the nucleus, exerting regulatory effects (40). Complementary studies evaluating these factors at the protein level, including quantification, localization, and phosphorylation, are warranted.

In the present study, we observed that the proportion of transitional follicles in the presence of CCL2 (both concentrations) was significantly higher relative to the control and the CCR2-antagonist groups. Furthermore, we observed an increase in the oocyte diameter in the CCL2-treated fragments and a decrease in the presence of the CCR2 antagonist. These findings suggest that CCR2 receptor activation stimulates the transition from primordial to primary follicles.

Next, we evaluated the role of CCR2 signaling on cell proliferation in ovarian cortex fragments, a key process in the transition from primordial to primary follicle, using two different markers (BrdU and Ki67). Both IHCs showed a higher proportion of proliferative transitional follicles in the CCL2_100_ group and reduced proliferation in the CCR2 antagonist group. These findings support the idea that activating the CCR2 chemokine receptor stimulates the transition from primordial to primary follicles. A higher proportion of cells were found to be BrdU-positive than Ki67-positive ones. This is likely because Ki-67 results only reflect a snapshot of the proliferation at the time of tissue fixation (50) rather than cumulative proliferation during the entire culture period, as is the case for BrdU labeling.

Given its role as an indirect marker of PI3K activity (10, 51) and as a marker of primordial follicle activation (7), we also assessed AKTp status in the feline ovarian cortex. Localization of the positive AKTp was observed either in the cytoplasm or in the nucleus or both in stromal cells, granulosa cells, and oocytes of primordial, transitional, primary, and secondary follicles. These results differ from studies in mice, where AKTp was observed only in the cytoplasm or oocyte membrane (52, 53). However, our results align with those observed in pig ovaries, where positive AKT was localized in the cytoplasm of oocyte and granulosa cells of primordial and primary follicles and vascular endothelial cells (47, 48). AKT phosphorylation and activation can be modulated by different ovarian growth factors, such as KitL, IGF1, and EGF (54–56). Interestingly, we observed a high staining intensity in the nucleus and cytoplasm of granulosa cells of primordial, transitional and primary follicles in the CCL2_10_ group, as expected since activation of the CCR2 receptor stimulates AKT phosphorylation (30, 57). These findings underline the concept that the CCR2 pathway stimulates follicular activation through AKTp, a known FOXO3 kinase necessary for its inactivation by exporting it from the nucleus to the cytoplasm, triggering primordial follicle activation (6–8, 10). Curiously, cellular localization of the positive staining within the different compartments (granulosa cells and oocyte only, or both) differs between primordial, transitional and primary follicles. Exclusively oocyte-positive staining was observed in primordial follicles in all the treatment groups. As the PI3K/Akt pathway plays an important role in many ovarian processes, through follicle establishment and activation, oocyte meiotic maturation, etc. (58) these findings suggest that CCR2 activation may act differently in primordial than in transitional and primary follicles, and further studies are needed to elucidate this. Engagingly, in the CCL2_10_ group we observed greater exclusively granulosa cells-positive staining localization in transitional follicles. Moreover, it is known that this pathway plays a key role in ovarian cancer pathogenesis by regulating many of the mechanisms involved (such as cell survival, growth, proliferation, angiogenesis, and metabolism).In rodents, it was shown that another chemokine/chemokine receptor system (CXCR4/SDF1) is involved in the follicle activation process, where an inhibitory role of the latter was observed in the primordial-to-primary transition (15), opposite to our present results. Conversely, our results support speculation made by Santos et al. using CCR2-/- mice (16), where they observed decreased follicular atresia and reduced follicular activation and proposed that the activation of the CCR2 receptor could stimulate the follicular activation process. However, their results do not demonstrate such an assumption since CCR2 expression was observed in oocytes from all classes of growing follicles except primordial follicles.

Notably, while it can be argued that the initial number of follicles in different developmental stages (primordial, transitional, primary, and secondary) in each ovarian fragment is unknown, a limitation of the current experimental model, the random tissue distribution among different treatment groups minimizes this bias. Additionally, differences in follicle activation exist among species, including variations in granulosa cell number and height, the percentage of transitional follicle stages, and specific molecular pathways that have been detailed in the manuscript (4, 28, 59).

The current study was performed using an *in vitro* model of the feline ovarian cortex. And like any model, it has its limitations by definition. However, it is unlikely that the results would be biased randomly in favor of the same side associated with the follicle activation process. Moreover, it was agreed that the culture system based on primordial follicles should begin with ovarian cortex tissue, as earlier human studies have shown a lower survival rate of isolated primordial follicle cultures compared to those from ovarian cortex tissue (60–66). Thus, supporting this culture system as a valid model to study follicle activation and primordial follicle biology. It is essential to emphasize that although the feline hypothalamic-pituitary-ovarian regulatory axis differs from that of humans, primordial follicle activation is a gonadotropin-independent process regulated by various autocrine and paracrine factors. Thus, this discrepancy in endocrine regulation between species is not relevant for studying this ovarian process. Moreover, the domestic cat is considered a valuable model for studying oocyte biology, as well as diverse infertility syndromes in women, due to the highly conserved reproductive mechanisms between the human and feline species (67) The domestic cat is considered a valuable model for studying oocyte biology, as well as diverse infertility syndromes in women, due to the highly conserved reproductive mechanisms between the human and feline species (67) This provides more analogous reproductive complexities to women than those found in rodents. Interestingly, cat oocytes share several characteristics with human oocytes (68, 69) in contrast to typical laboratory mouse models. These similarities include the diameter of the oocyte proper and the germinal vesicle, the time to reach the metaphase II stage of meiosis in culture, and a nuclear configuration with a small nucleolus and fibrillar chromatin. In addition to serving as a valuable model species for biomedical research, utilizing the feline model also has the potential to expand knowledge of feline reproduction, which is crucial for both veterinary care and the conservation of endangered felids.

## Conclusion

Our results demonstrate a direct effect of the chemokine CCL2 and a role for the CCR2/CCL2 system in the ovarian cortex, suggesting that CCR2 receptor signaling in the ovarian cortex may regulate events crucial for promoting the transition from primordial to primary follicles. This could have potential therapeutic applications by explicitly focusing on targeting CCR2 within the ovary in fertility treatments, and further studies are warranted.

## Data Availability

The original contributions presented in the study are included in the article/**Supplementary Material**. Further inquiries can be directed to the corresponding author.
