# Supplementary material for: Novel direct effect of CCR2 receptor on follicle activation process
Source: Front Endocrinol (Lausanne). 2025 Aug 1;16:1613270. doi: 10.3389/fendo.2025.1613270 (PMC12353731; doi:10.3389/fendo.2025.1613270)
Supplement: Supplementary file 1 [file Image1.pdf]

Supplemental Figure 1

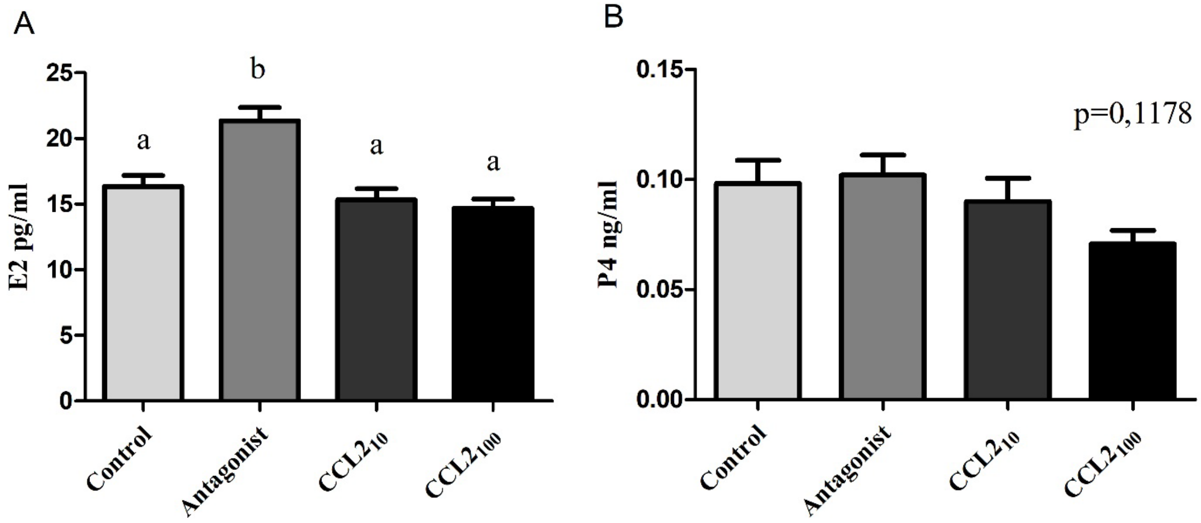

Supplemental Figure 1. Low levels of E2 and P4 from the culture medium of feline ovarian cortex fragments cultured 48 h in the presence of different treatments (Control, CCR2 Antagonist, CCL210, and CCL2100). Values are mean ± SEM (pg/ml) of E2 (panel A; n=9/treatment) and P4 (panel B; n=9/treatment. Different letters represent significant group differences (ANOVA; $p<0.05$). Detection limit: E2 (<10 pg/mL): P4 (<0.05 ng/mL).
