# Supplementary material for: Novel direct effect of CCR2 receptor on follicle activation process
Source: Front Endocrinol (Lausanne). 2025 Aug 1;16:1613270. doi: 10.3389/fendo.2025.1613270 (PMC12353731; doi:10.3389/fendo.2025.1613270)
Supplement: Supplementary file 2 [file Table1.docx]

**Supplementary Table 1.** Fragment area and follicle density among different treatment groups

|  | **Control** | **CCR2 Antagonist** | **CCL2 _10_** | **CCL2 _100_** |
| --- | --- | --- | --- | --- |
| **Fragment Area** (mm2) | 247630 ± 30451 | 264036 ± 41999 | 270663 ± 29834 | 236517 ± 22664 |
| **Follicle Density**  (follicles/mm2) | 22.32 ± 4.26 | 26.02 ± 4.09 | 22.64 ± 2.67 | 23.75 ± 3.45 |

- Values are the mean ± SEM. No significant differences were observed among groups.
