# Supplementary material for: Novel direct effect of CCR2 receptor on follicle activation process
Source: Front Endocrinol (Lausanne). 2025 Aug 1;16:1613270. doi: 10.3389/fendo.2025.1613270 (PMC12353731; doi:10.3389/fendo.2025.1613270)
Supplement: Supplementary file 3 [file Table2.docx]

| **Supplementary Table 2.** Forward and reverse primers sequences | | | | |
| --- | --- | --- | --- | --- |
| **Gen**  **Symbol** | **Forward (5’-3’)** | **Reverse (5’-3’)** | **Target sequence accession number** |  |
| ***18s*** | CGGCTACCACATCCAAGGAA | GGGCCTCGAAAGAGTCCTGT | XR_002740181.1 |  |
| ***GAPDH*** | GCCATCAATGACCCCTTCAT | GCCGTGGAATTTGCCGT | XM_006933438.4 |  |
| ***FOXO3*** | GACCCCTTGATGTCTCAGGC | TGACAGGAGATTGCTGCTGG | XM_003986453.6 |  |
| ***mTOR*** | GCTGTACGACCTGAGCCGTC | GTGGCGGACAAACTGGGTCA | XM_003989489.4 |  |
|  |  |  | XM_045035086.1 |  |
|  |  |  | XM_045035087.1 |  |
|  |  |  | XM_045035089.1 |  |
| ***PIK3Ca*** | CAATCGGTGACTGTGTGGGA | ATGGCTGCATCGTATATTTCGC | XM_023260376.2 |  |
|  |  |  | XM_019840086.3 |  |
| ***KIT*** | GATAGCACCAATCATATTTATTCCAAC | CCACAGAATTGATCCGCAC | NM_001009837.3 |  |
| ***KITL*** | AGAAGACACAAACTTGGATTGTCAC | CACATCATCAGTCACACGGTTC | XM_045060759.1 |  |
|  |  |  | XM_045060760.1  XM_045060758.1  XM_045060757.1 |  |
|  |  |  | XM_045060761.1 |  |
| ***AKT1*** | CCCCGCTCAACAACTTCTCT | AAACTCGTTCATGGTCACGC | NM_001322435.1 |  |

*GAPDH*: Glyceraldehyde-3-Phosphate Dehydrogenase; *FOXO3*: Forkhead Box O3; *mTOR*: Mechanistic Target Of Rapamycin Kinase; *PIK3Ca*: Phosphatidylinositol-4,5-Bisphosphate 3-Kinase Catalytic Subunit Alpha; *KIT*: KIT Proto-Oncogene, Receptor Tyrosine Kinase; KITL: KIT Ligand; *AKT1*: AKT Serine/Threonine Kinase 1.
